# New Jersey State Veterinary Society

**Published:** 1886-10

**Authors:** Wm. Herbert Lowe

**Affiliations:** Secretary


### NEW JERSEY STATE VETERINARY SOCIETY.

The eighth regular meeting of this association was held at Long Branch, Thursday, August 12th, 1886. The President, Dr. Wm. B. E. Miller, of Camden, occupied the chair and called the meeting to order.

Dr. Lowe announced that he had just received a letter from Dr. Liautard, in which he said that he was obliged to excuse himself on account of an unexpected professional duty. The professor expressed regret at not being able to meet "many as former pupils, all as good workers in the profession."

Dr. Lowe also read letters from Dr. E. M. Hunt, Secretary of the New Jersey State Board of Health, Dr. F. S. Billings and others, in which they expressed a sincere desire for the welfare of the society, and regretted not being able to be present.

President Miller delivered an excellent address on Veterinary Legislation, showing how important it is that the Legislature should grant the profession still greater protection than has heretofore been accorded it by the act under which the Association is incorporated.

The members of the society had a lengthy discussion over the practice, now quite common with insurance companies, of employing their own veterinarians to look after horses that are insured by them. This method was condemned and a resolution was adopted not to treat horses that are insured in these companies, or to meet such veterinary surgeons in consultation.

The system of agricultural and other colleges having veterinary departments which are filled by but one professor, graduating young men as veterinary surgeons was generally condemned. It was the opinion of the members present that justice could not possibly be done to veterinary students at such colleges, and that it was an imposition upon the student, professor and the public in general.

The next regular meeting will be held at Trenton in December.

WM. HERBERT LOWE, D. V. S., *Secretary.*

